# Evaluation de deux tests de diagnostic antigénique du COVID-19: *BIOSYNEX^®^ COVID-19 Ag BSS* et *BIOSYNEX^®^ COVID-19 Ag+ BSS* comparés à la *PCR AmpliQuick^®^ SARS-CoV-2*

**DOI:** 10.11604/pamj.2021.39.228.30752

**Published:** 2021-08-06

**Authors:** Guy Pascal Ngaba, Ginette Claude Mireille Kalla, Jules Clément Nguedia Assob, Abdel Jelil Njouendou, Christian Nelly Jembe, Emile Télesphore Mboudou, François-Xavier Mbopi-Keou

**Affiliations:** 1 Faculté de Médecine et des Sciences Pharmaceutiques, Université de Douala, Douala, Cameroun,; 2 Hôpital Gynéco-Obstétrique et Pédiatrique de Douala, Douala, Cameroun,; 3 Faculté de Médecine et des Sciences Biomédicales, Université de Yaoundé I, Yaoundé, Cameroun,; 4 Faculté des Sciences de la Santé, Université de Buea, Buea, Cameroun,; 5 Ministère de la Santé publique, District de santé de Deïdo, Douala, Cameroun,; 6 Institut pour le Développement de l'Afrique (The-IDA), Yaoundé, Cameroun

**Keywords:** Evaluation, AmpliQuick^®^ SARS-CoV-2, BIOSYNEX^®^COVID-19 Ag BSS, BIOSYNEX^®^COVID-19 Ag+ BSS, Douala, Cameroun, Evaluation, AmpliQuick^®^ SARS-CoV-2, BIOSYNEX^®^ COVID-19 Ag BSS, BIOSYNEX^®^ COVID-19 Ag+ BSS, Douala, Cameroun

## Abstract

**Introduction:**

la pandémie à COVID-19 pose des problèmes de diagnostic biologique qui restent d´actualité dans les pays à faible revenu en général et au Cameroun en particulier. Les tests rapides détectant l´antigène du virus SARS-CoV-2 fiables se présentent comme une alternative importante. L´objectif de notre étude était d´évaluer les performances diagnostiques des tests de diagnostic rapide BIOSYNEX^®^COVID-19 Ag BSS et BIOSYNEX^®^COVID-19 Ag+ BSS comparés entre eux, puis comparés au test PCR AmpliQuick^®^ SARS-CoV-2.

**Méthodes:**

une étude transversale et comparative a été menée du 27 avril au 29 mai 2021 dans la ville de Douala au Cameroun. Les échantillons étaient constitués de prélèvements nasopharyngés reçus au laboratoire de biologie moléculaire de l'Hôpital Gynéco-Obstétrique et Pédiatrique de Douala. Les paramètres sociodémographiques (âge, profession (footballeurs, voyageurs, autres), statut matrimonial, nationalité), comorbidité et statut connu du COVID-19, ont été enregistrés sur les sites de collecte. Les principaux sites de collecte étaient le District de santé de Deïdo et l'Hôpital Gynéco-Obstétrique et Pédiatrique de Douala. Nous avons effectué le diagnostic du COVID-19 en utilisant les tests de diagnostic rapide BIOSYNEX^®^ COVID-19 Ag BSS et BIOSYNEX^®^ COVID-19 Ag+ BSS que nous avons d´abord comparé entre eux, puis nous les avons comparés au test PCR AmpliQuick^®^SARS-CoV-2 sur chaque échantillon. L'analyse statistique des données a été réalisée à l'aide des logiciels Microsoft Excel et SPSS version 17. Pour déterminer la sensibilité des deux tests de diagnostic rapide, le modèle bayésien de classe latente a été réalisé sur la médiane avec un intervalle de confiance à 95 %, et p<0,05 comme seuil de significativité. Une autorisation éthique a été demandée et obtenue auprès du Comité d'éthique Institutionnel (CEI) de l'Université de Douala.

**Résultats:**

un total de 1813 participants a été inclus dans cette étude, avec une prédominance d'hommes (1226, 68,68 %) et la tranche d'âge la plus représentée était celle des 31 à 40 ans (568, 31,33 %). La plupart des participants étaient mariés (888, 53,46%) et seuls quelques-uns avaient un statut COVID-19 connu (75, 5,47%). Les prévalences de COVID-19 retrouvées avec les deux tests de diagnostic rapide étaient de 2,03 et 2,17 pour BIOSYNEX^®^ COVID-19 Ag BSS et BIOSYNEX^®^COVID-19 Ag + BSS respectivement. Une sensibilité de 94,1% a été retrouvée pour le test BIOSYNEX^®^ COVID-19 Ag BSS tandis que pour le test BIOSYNEX^®^ COVID-19 Ag + BSS, la sensibilité était de 87,5%. La spécificité était de 98,9% et 98,7% pour le test BIOSYNEX^®^ COVID-19 Ag + BSS et BIOSYNEX^®^ COVID-19 Ag BSS respectivement par rapport à AmpliQuick^®^ SARS-CoV-2. Le test BIOSYNEX^®^ COVID-19 Ag + BSS a montré une valeur prédictive négative de 99.9% par rapport au test BIOSYNEX^®^ COVID-19 Ag BSS. Un accord de 99,9% a été retrouvé entre les tests BIOSYNEX^®^ COVID-19 Ag BSS et BIOSYNEX^®^ COVID-19 Ag + BSS. **Conclusion:** les tests de diagnostic rapide BIOSYNEX^®^ COVID-19 Ag + BSS et BIOSYNEX^®^ COVID-19 Ag BSS peuvent être utilisés pour le diagnostic du SARS-CoV-2 et peuvent être d´un apport important dans le dépistage de masse et le dépistage en zones reculées.

## Abstract (en)

**Introduction:**

the COVID-19 pandemic causes biological diagnostic problems that remain relevant in low-income countries in general and in Cameroon in particular. Rapids tests that reliably detect SARS-CoV-2 virus antigen present themselves as an important alternative in several contexts. The objective of our study was to evaluate the diagnostic performance of two rapid diagnostic tests BIOSYNEX^®^ COVID-19 Ag BSS and BIOSYNEX^®^ COVID-19 Ag + BSS, compared to each other and to the AmpliQuick^®^ SARS-CoV-2 PCR test.

**Methods:**

a cross-sectional and comparative study was carried out from April 27 to May 29, 2021 in the city of Douala in Cameroon. The samples consisted of nasopharyngeal swabs received at the molecular biology laboratory of the Douala Gyneco-obstetric and pediatric hospital, whatever their origin. The socio-demographic parameters (age, profession, football players, travelers, others), marital status, nationality), comorbidity and known status of COVID-19, were recorded on the collection sites. The main collection sites were the Deïdo Health District and the Douala Gyneco-Obstetric and Pediatric Hospital. We performed the diagnosis of COVID-19 using the rapid diagnostic test (RDT) BIOSYNEX^®^ COVID-19 Ag BSS and RDT BIOSYNEX^®^ COVID-19 Ag + BSS compared to each other and to the AmpliQuick^®^ SARS-CoV-2 polymerase chain reaction (PCR) test on each sample. Statistical analysis of the data was performed using Microsoft Excel and SPSS version 17 software. To determine the sensitivity of the two RDTs, the Bayesian latent class model was performed on the median with a 95% confidence interval with p<0.05 as the significant level. An ethical clearance was sought and obtained from the University of Douala Institutional Ethics Committee.

**Results:**

a total of 1813 participants were included in our study, with a predominance of men (1226, 68.68 %) and the most represented age group was that of 31 to 40 years (568, 31.33 %). Most of the participants were married (888, 53.46%) and only a few had a known COVID-19 status (75, 5.47%). The two rapid tests on our study population show much closed COVID-19 prevalence values, respectively 2.03 for BIOSYNEX^®^ COVID-19 Ag BSS and 2.17 for BIOSYNEX^®^ COVID-19 Ag + BSS. RDT BIOSYNEX^®^ COVID-19 Ag + BSS showed higher sensitivity 94.1% vs. 87.5% for RDT BIOSYNEX^®^ COVID-19 Ag BSS with almost identical specificity 98.9% for RDT BIOSYNEX^®^ COVID-19 Ag + BSS vs. 98.7% for RDT BIOSYNEX^®^ COVID-19 Ag BSS compared to AmpliQuick^®^ SARS-CoV-2. BIOSYNEX^®^ COVID-19 Ag + BSS RDT showed a negative predictive value of 99.9% compared to BIOSYNEX^®^ COVID-19 Ag BSS RDT. There is a 99.9% agreement between the RDT BIOSYNEX^®^ COVID-19 Ag BSS and the RDT BIOSYNEX^®^ COVID-19 Ag + BSS. **Conclusion**: the RDT BIOSYNEX^®^COVID-19 Ag + BSS and RDT BIOSYNEX^®^ COVID-19 Ag BSS can be used for the diagnosis of SARS-CoV-2 and can have an important contribution in the context of mass screenings and screening in remote areas.

## Introduction

Le nouveau coronavirus 19, responsable de la maladie à coronavirus 19 reste une menace à l´échelle mondiale, malgré le début de la vaccination dans un grand nombre de pays. A la différence des autres continents (6-39%), la prévalence est restée faible dans les pays africains en général et en Afrique centrale en particulier (autour de 2%). Au Cameroun, après une deuxième vague avec un pic au mois de mars 2021, on observe une décroissance marquée du nombre de cas et de décès confirmée par la décroissance du taux de positivité des tests PCR [1]. Le diagnostic biologique reste l´un des piliers de la maitrise de cette pandémie [2, 3]. L´accès au diagnostic par PCR a beaucoup évolué depuis le début de la pandémie dans les pays développés [4, 5] et n´a que modestement progressé dans les pays à faible revenu en général et au Cameroun en particulier [5]. La stratégie choisie est le dépistage de masse par l´utilisation des tests de diagnostic rapide (TDR).

Malgré leur faible sensibilité par rapport à la biologie moléculaire, ces tests ont l´avantage d´une utilisation facile, et ne nécessitent pas une expertise particulière. Pour certains, ils peuvent être réalisés non seulement sur les prélèvements nasopharyngés, mais également sur les échantillons salivaires ou nasaux [3-5]. La nécessité de procéder à une recherche rapide de cas, a poussé au développement des TDR basés essentiellement sur les techniques immunochromatographiques à partir des prélèvements nasopharyngés et actuellement même sur les prélèvements salivaires [3,5-8]. Selon l´Organisation Mondiale de la Santé, le test de référence reste la Réaction Polymérase en Chaine (PCR) [2] mais compte tenu de la complexité de la mise en œuvre de cette technique dans les pays à faible revenu, des difficultés à assurer l´approvisionnement constant et de l´absence de garantie d´un accès gratuit permanent à cette technique, il est important de proposer des TDR qui soient fiables, suffisamment sensibles et spécifiques pour réduire au maximum les risques de passer à côté d´un cas positif [2]. Les défis importants que constituent les grands regroupements des populations au cours des compétitions sportives [9] et l´accès très limité aux vaccins obligent à augmenter l´accès à des tests fiables autres que la PCR. Ces TDR donnent des résultats en 15 minutes et permettent de réduire assez significativement la circulation virale [2-5]. Il existe actuellement au Cameroun très peu de tests de diagnostic rapide et pour la grande majorité d´entre eux, ils n´ont pas fait l´objet d´une évaluation avant leur entrée sur le territoire. Nous avons évalué les performances de 2 tests de diagnostic rapide antigéniques à savoir le *BIOSYNEX^®^COVID-19 Ag BSS* et le *BIOSYNEX^®^ COVID-19 Ag+ BSS* [10] comparés d´abord entre eux, puis au test PCR AmpliQuick^®^ SARS-CoV-2 [10].

## Méthodes

**Lieu et type d´étude**: l´étude a été menée au laboratoire de biologie moléculaire de l'Hôpital Gynéco-Obstétrique et Pédiatrique de Douala (HGOPED) pour les échantillons en provenance du District de Santé de Deïdo et de l´Hôpital Gynéco-Obstétrique et Pédiatrique de Douala. Il s´est agi d´une étude transversale et comparative du 27 avril au 29 mai 2021 dans la ville de Douala au Cameroun.

**Population étudiée**.

**Population cible**: toutes les personnes reçues au District de Santé de Deïdo et à l'Hôpital Gynéco-Obstétrique et Pédiatrique de Douala (HGOPED) qui ont sollicité un test COVID-19.

**Critères d'inclusion**: toutes les personnes, tout âge confondu, reçues au District de Sanité de Deïdo et à l'Hôpital Gynéco-Obstétrique et Pédiatrique de Douala (HGOPED) pendant la période d'étude et qui ont sollicité un test COVID-19.

**Critères de non inclusion**: patients gravement malades.

**Collecte de données**: les échantillons consistaient en des écouvillons nasopharyngés. Deux échantillons pour les tests de diagnostic rapide réalisés sur les sites de collecte et un écouvillon acheminé dans son milieu de transport au laboratoire de biologie moléculaire pour la PCR AmpliQuick^®^ SARS-CoV-2 [10]. À l'aide d'un questionnaire, des informations sur les paramètres sociodémographiques (âge, profession (footballeurs, voyageurs, statut matrimonial, nationalité, autres), comorbidité et statut connu de COVID-19 ou non ont été enregistrés sur les sites de collecte. Il s´agissait de participants adultes consentants (et des enfants et adolescents dont les parents ont donné leur consentement éclairé). Chaque échantillon reçu a été systématiquement analysé par PCR AmpliQuick^®^ SARS-CoV-2 et par les TDR *BIOSYNEX^®^ COVID-19 Ag BSS* et *BIOSYNEX^®^ COVID-19 Ag+ BSS*. La valeur minimale du seuil de cycle (Ct) pour ces gènes a été retenue pour l'analyse statistique. Les résultats de la réaction en chaîne de polymése de transcription inverse (RT-PCR) ont été considérés comme positifs si un gène spécifique du SARS-CoV-2 pouvait être amplifié avec moins de 33 cycles. En l'absence de SARS-CoV-2 amplification génique, la valeur Ct était de 0 et le résultat de la RT-PCR était négatif.

**Description des tests**: *BIOSYNEX^®^ COVID-19 Ag* et *BIOSYNEX^®^ COVID-19 Ag+ BSS* sont des autotests en Kit de 5 tests antigéniques pour la détection du SARS-CoV-2, donnant des résultats respectivement en 15 minutes et 10 minutes sur les prélèvements nasals [10]. La différence majeure étant la durée de rendu des résultats. La PCR AmpliQuick^®^ SARS-CoV-2 quant à elle permet la détection de 3 gènes du SARS-CoV-2, (2 gènes viraux: le gène RdRp ainsi que le gène E; et un gène humain RNaseP servant de contrôle procédural) [10].

**Analyses statistiques**: nous avons évalué la technique la plus avantageuse en termes de performances diagnostiques de précision. Les paramètres de performance analytique (relatifs à la qualité de détection du marqueur d'intérêt) ont été déterminés. L'analyse statistique des données a été réalisée à l'aide des logiciels Microsoft Excel et IBM^®^ SPSS version 20 (Chicago, Illinois, États-Unis). La sensibilité (Se), la spécificité (Sp), la valeur prédictive positive (VPP) et la valeur prédictive négative (VPN) des deux TDR ont été calculées selon des méthodes standard. Pour l'analyse primaire, la sensibilité (taux de vrais positifs) a été définie comme la probabilité que le résultat du TDR soit positif lorsque la PCR AmpliQuick^®^ SARS-CoV-2 a confirmé la présence de traces d´acides nucléiques du virus; tandis que la spécificité (taux de vrais négatifs) a été définie comme la probabilité que le résultat du TDR soit négatif lorsque les traces d´acide nucléiques viral n´ont pas été révélés par la PCR AmpliQuick^®^ SARS-CoV-2. La VPP était la probabilité que la PCR AmpliQuick^®^ SARS-CoV-2 ait confirmé la présence d´acide nucléique viral lorsque le TDR était positif, et la VPN était la probabilité que la PCR AmpliQuick^®^ SARS-CoV-2 ait confirmé l´absence d´acide nucléique viral lorsque le TDR était négatif. En supposant l´inexistence d´un test de référence parfait, les résultats des trois tests ont été soumis au modèle bayésien de classe latente. Cette analyse était effectuée à l´aide d´une application Web décrite par Lim *et al*. [11] et basée sur les modèles bayésiens de classe latente (LCM). Ce modèle de statistique diagnostique a été utilisé pour déterminer la performance (sensibilité, spécificité, VPP et VPN) des deux TDR en utilisant la PCR AmpliQuick^®^ SARS-CoV-2 comme étalon imparfait avec l'aide d'une interface simplifiée de trois tests dans un modèle à une population (modèle Walter et Irwig) [11]. Les paramètres diagnostiques étaient exprimés sous forme de médiane associés d´intervalle de confiance à 95%. La valeur p<0,05 était considérée comme niveau de significativité. L´analyse de concordance entre les tests a été réalisée en calculant le paramètre Fleiss' *kappa (κ)* (Agreement). Ce paramètre a été interprété en utilisant les seuils décrits par Landis and Koch 1977 [12].

**Considérations éthiques**: une autorisation éthique a été demandée et obtenue auprès du Comité d'éthique Institutionnel (CEI) de l'Université de Douala (2739IEC-UD/US2021M). Ce CEI est habilité par le Comité National d'Ethique du Cameroun à évaluer toutes les recherches sur les projets de santé humaine menées dans les institutions de recherche et les institutions hospitalières basées dans la Région du Littoral. Chaque participant a consenti à faire partie de l'étude. L'anonymat a été conservé.

## Résultats

**Caractéristiques sociodémographiques de la population étudiée**: le Tableau 1 présente les caractéristiques sociodémographiques de la population. Sur un total de 1813 participants, 1298 étaient des camerounais (71,59%) contre 515 d´autres nationalités (28,41%), la plupart âgés de 31 à 40 ans (568, 31,33%). Le sexe masculin dominait (68,68 %) et ils étaient pour la plupart mariés (1226, 53,46%). Seuls quelques participants (75, 5,41%) connaissaient leur statut COVID-19. Les prélèvements nasopharyngés constituaient la grande majorité (1836, 99,83%).

**Tableau 1:** caractéristiques sociodémographiques de la population étudiée

Variables	Fréquence	Pourcentage
**Nationalité**		
Camerounaise	1298	71,59
Autres	515	28,41
Total	1813	
**Age**		
0-17	53	2,92
18-30	350	19,31
31-40	568	31,33
41-50	482	26,59
51-60	229	12,63
61-70	103	5,68
71+	28	1,54
Total	1813	
**Sexe**		
Masculin	1226	68,68
Féminin	559	31,32
Total	1785	
**Statut matrimonial**		
Marié	888	53,46
Célibataire	743	44,73
Divorcé	30	1,81
Total	1661	
**Statut COVID-19 connu**		
Oui	75	5,47
Non	1295	94,53
Total	1370	
**Type d´échantillon**		
Nasopharyngé	1836	99,83
Nasal	2	0,12
Gorge	1	0,05
Total	1839	

**Performance des tests**: le Tableau 2 présente les performances diagnostiques des TDR *BIOSYNEX^®^ COVID-19 Ag BSS* et *BIOSYNEX^®^ COVID-19 Ag+ BSS*/AmpliQuick^®^ SARS-CoV-2 réalisés au cours de la même période et révèle que l´utilisation des 2 tests de diagnostic rapide sur notre population d´étude montre des prévalences du COVID-19 très proches, 2,03 (IC95%= [1,04-3,01]) et 2,17 (IC95%= [1,14-3,17]) respectivement. Les deux TDR ont un niveau de précision quasi identique de respectivement 98,3% (Tableau 3) pour *BIOSYNEX^®^ COVID-19 Ag BSS* et 98,7% pour *BIOSYNEX^®^ COVID-19 Ag+ BSS* comparé au gold standard (AmpliQuick^®^ SARS-CoV-2). Le TDR *BIOSYNEX^®^ COVID-19 Ag+ BSS* a montré une sensibilité plus élevée de 94,1% contre 87,5% pour le TDR *BIOSYNEX^®^ COVID-19 Ag BSS* avec une spécificité quasi identique 98,9% pour *BIOSYNEX^®^ COVID-19 Ag+ BSS* contre 98.7% pour *BIOSYNEX^®^ COVID-19 Ag BSS* par rapport à AmpliQuick^®^ SARS-CoV-2.

**Tableau 2:** prévalence des tests *BIOSYNEX^®^ COVID-19 Ag BSS, BIOSYNEX^®^ COVID-19 Ag+ BSS* et AmpliQuick SARS-CoV-2

Tests	Nombre de tests effectués	Résultats positifs	Prévalence(%)	95% IC
*BIOSYNEX^®^COVID-19 Ag BSS*	789	16	2,03	[1,04-3,01]
*BIOSYNEX^®^COVID-19 Ag+ BSS*	789	17	2,15	[1,14-3,17]
AmpliQuick	1810	35	1,93	[1,3-2,57]

IC: intervalle de confiance

**Tableau 3:** performances diagnostiques comparées des deux tests antigéniques TDR *BIOSYNEX^®^ COVID-19 Ag+ BSS* et TDR *BIOSYNEX^®^ COVID-19 Ag BSS* Versus AmpliQuick (AmpliQuick comme standard)

Résultats	Résultat AmpliQuick (n)		Total	Se	Sp	VPP	VPN	LR+	LR-	Exactitude
*BIOSYNEX^®^COVID-19 Ag BSS*	Positif	Négatif	(n)	(%)	(%)	(%)	(%)			(%)
BIOSYNEX^®^COVID-19 Ag BSS										
Positif	14	2	16	87,5	98,6	56	99,7	6130	12,7	98,3
Négatif	11	760	771							
Total	25	762	787							
*BIOSYNEX^®^COVID-19 Ag+ BSS*										
Positif	16	1	17	94,1	98,8	64	99,9	8050	6	98,7
Négatif	9	761	770							
Total	25	762	787							

TDR1: TDR *BIOSYNEX^®^COVID-19 Ag BSS* ; TDR2: TDR *BIOSYNEX^®^COVID-19 Ag+ BSS* ; Se: sensibilité; Sp: spécificité ; VPP: valeur prédictive positive; VPN: valeur prédictive négative; LR+: rapport de vraisemblance positif; LR-: rapport de vraisemblance négatif

Le TDR *BIOSYNEX^®^ COVID-19 Ag BSS* a montré une valeur prédictive positive de 56% contre 64% pour le TDR *BIOSYNEX^®^ COVID-19 Ag+ BSS* par rapport à AmpliQuick^®^ SARS-CoV-2. Le TDR *BIOSYNEX^®^ COVID-19 Ag+ BSS* a montré une valeur prédictive négative de 99,9% par rapport au TDR *BIOSYNEX^®^ COVID-19 Ag BSS* (Tableau 3). En ignorant l´existence d´un étalon standard parfait, l´analyse du Modèle Bayésien de classe latente a révélé d´excellents paramètres de performance diagnostique pour le TDR *BIOSYNEX^®^ COVID-19 Ag+ BSS* (Tableau 4). La sensibilité et la spécificité de ce test étaient de 98,3% (IC95%= 82,8-100), et 100% (IC95%=99,7-100) respectivement, alors que ces paramètres étaient de 92,9% (IC95% (74,1-99,3), et 98,8% (IC95% 97,9-99,4) pour la PCR AmpliQuick^®^ SARS-CoV-2 et 87,2% (IC95% (66,3-97,4), et 99,9% (IC95% (99,4-100) pour le TDR *BIOSYNEX^®^COVID-19 Ag BSS*. En outre, la puissance du TDR *BIOSYNEX^®^ COVID-19 Ag+ BSS* à discriminer les faux négatifs s´est prononcée avec une VPN de 100%, contre 99,8% et 99,7% en ce qui concerne la PCR et le TDR *BIOSYNEX^®^ COVID-19 Ag BSS*.

**Tableau 4:** performances diagnostiques des deux TDR *(TDR BIOSYNEX^®^ COVID-19 Ag BSS* et TDR *BIOSYNEX^®^ COVID-19 Ag+ BSS)* et PCR AmpliQuick révélées par l´analyse du modèle Bayésien de classe latente

Paramètres	Modèle Bayésien de classe latente 95%(IC)
PCR Ampliquick	
Sensibilité	92,9(74,1-99,3)
Spécificité	98,8(97,9-99,4)
VPP	63,2(43,5-80,6)
VPN	99,8(99,3-100)
TDR *BIOSYNEX^®^COVID-19 Ag BSS*	
Sensibilité	87,2(66,3-97,4)
Spécificité	99,9(99,4-100)
VPP	93,0 (74,0-99,7)
VPN	99,7(99,2-100)
TDR *BIOSYNEX^®^COVID-19 Ag+ BSS*	
Sensibilité	98,3(82,8-100)
Spécificité	100 (99,7-100)
VPP	98,7(85,7-100)
VPN	100(99,6-100)

VPP: valeur prédictive positive ; VPN: valeur prédictive négative

L´analyse de concordance entre les tests a révélé (Tableau 5) l´existence d´un accord presque parfait entre les deux TDR (κ=0,907). Cependant, une concordance substantielle était observée entre le TDR *BIOSYNEX^®^ COVID-19 Ag BSS* vs PCR (κ=0,674) d´une part et le TDR *BIOSYNEX ^®^COVID-19 Ag+ BSS* vs PCR d´autre part (κ=0,756).

**Tableau 5:** mesures de concordance entre les deux TDR (tests antigéniques TDR *BIOSYNEX^®^ COVID-19 Ag BSS*, TDR *BIOSYNEX^®^ COVID-19 Ag+ BSS)* et la PCR AmpliQuick

Tests	Kappa(κ)	p-value	Interprétation
TDR *BIOSYNEX^®^COVID-19 Ag+ BSS* vs TDR *BIOSYNEX^®^COVID-19 Ag BSS*	0,907	<0,001	Accord quasi parfait
TDR *BIOSYNEX^®^COVID-19 Ag+ BSS* vs AmpliQuick	0,756	<0,001	Accord substantiel
AmpliQuick vs TDR *BIOSYNEX^®^COVID-19 Ag BSS*	0,674	<0,001	Accord substantiel

La statistique κ s´interprète comme suit: 0 = concordance équivalente au hasard; 0,10-0,20 = faible concordance ; 0,21-0,40 = concordance équitable ; 0,41-0,60 = concordance modérée ; 0,61-0,80 = concordance substantielle ; 0,81-0,99 = concordance presque parfaite; et 1,00 = concordance parfaite. Les valeurs négatives indiquent que l´accord observé est pire que ce à quoi on pourrait s´attendre par hasard [11].

## Discussion

A notre connaissance, il s'agit de la première étude évaluant la performance des TDR *BIOSYNEX^®^ COVID-19* en Afrique. On a noté dans cette population, une proportion plus importante des personnes mariées et de sexe masculin, donc à risque d´entrer en contact avec des personnes dans leurs entourages, ceci confirme la notion de risque de contagion avec un parent proche positif au COVID-19, et la propagation de l'épidémie [13]. La problématique du diagnostic biologique du SARS-CoV-2 demeure dans les pays à faible revenu en général et au Cameroun en particulier. Le diagnostic moléculaire reste très limité, et ce, même dans les grandes villes. La perspective de disposer de résultats en moins de 20 minutes [2] met en lumière le rôle des TDR antigéniques pour le dépistage du SARS-CoV-2. Ceci peut pallier aux grands délais de remise des résultats des PCR qui en l´occurrence prennent plus de temps (3 à 24 heures) [6]. Ces délais sont susceptibles de retarder la prise en charge des cas et favoriser la propagation en communauté de la pandémie à COVID-19 [2,3,5]. Outre la réduction des délais de 1h 45 minutes pour l'AmpliQuick^®^ SARS-CoV-2 à 15 et 10 minutes pour les 2 tests rapides antigéniques (TDR *BIOSYNEX^®^ COVID-19 Ag BSS* et *BIOSYNEX^®^ COVID-19 Ag+ BSS* respectivement), nous avons noté une grande souplesse d´utilisation des tests rapides qui ne nécessitent pas une expertise particulière. Des prévalences de 2,03 % et 2,15 % ont été obtenues respectivement pour le TDR *BIOSYNEX^®^ COVID-19 Ag BSS* et le TDR *BIOSYNEX^®^ COVID-19 Ag+ BSS* tandis que l´AmpliQuick^®^ SARS-CoV-2 présentait une prévalence de 1,93% pour une population testée plus importante.

A l´évaluation, des paramètres diagnostiques des deux TDR (*BIOSYNEX^®^ COVID-19 Ag BSS* et *BIOSYNEX^®^ COVID-19 Ag+ BSS*), on a noté que les deux tests répondent aux exigences minimales de performance des tests rapides selon l´OMS, qui n´autorisent que des TDR qui ont une sensibilité supérieure ou égale à 80% et une spécificité supérieure ou égale à 97% [2]. Nous avons également des niveaux d´accord entre les deux TDR *BIOSYNEX^®^ COVID-19 Ag BSS* et *BIOSYNEX^®^ COVID-19 Ag+ BSS* qui sont proches de la perfection, comparés entre eux et qui ont un accord comparé à la PCR AmpliQuick^®^ SARS-CoV-2. De plus les valeurs diagnostiques de ces deux TDR concordent avec celles du fabriquant. Nous avons noté toutefois une supériorité du TDR *BIOSYNEX^®^ COVID-19 Ag+ BSS* sur le TDR *BIOSYNEX^®^ COVID-19 Ag BSS* en termes de sensibilité et spécificité. En plus de cet avantage le TDR *BIOSYNEX^®^ COVID-19 Ag+ BSS* permet d´obtenir les résultats en 10 minutes comparé au TDR *BIOSYNEX^®^COVID-19 Ag BSS* qui requiert 15 minutes.

**Limite de l´étude**: cette étude a présenté comme limite l´impossibilité de recontacter les participants pour avoir leur avis sur l´utilité des résultats des TDR obtenus. Il était également difficile d´analyser les données sur les signes cliniques que présentaient les participants au regard du faible niveau de complétude de cette variable.

**Avantages**: nous avons évalué un nombre important d´échantillons donnant plus de puissance statistique à notre travail de recherche.

## Conclusion

Les tests de diagnostic rapide *BIOSYNEX^®^ COVID-19 Ag+ BSS* et *BIOSYNEX^®^ COVID-19 Ag BSS* peuvent être utilisés pour le diagnostic du SARS-CoV-2 dans notre contexte et peuvent être d´un apport important dans le cadre des dépistages de masse et des dépistages en zones reculées dépourvues de moyens technologiques. Ainsi, l´obtention de résultats fiables et rapides par les TDR peuvent contribuer au contrôle de la pandémie à COVID-19.

### Etat des connaissances sur le sujet

- La pandémie de COVID-19 n'est pas encore résolue;
- La pandémie actuelle de COVID-19 a renforcé la position centrale des tests de diagnostic rapide dans le contrôle des épidémies dans le monde entier;
- La PCR en temps réel est donc recommandée pour détecter l'infection par le SARS-CoV-2 si une réponse adéquate doit être assurée.

### Contribution de notre étude à la connaissance

- La sensibilité et la spécificité des TDR BIOSYNEX^®^ COVID-19 Ag+ BSS et BIOSYNEX ^®^COVID-19 Ag BSS répondent aux exigences de performance de l´OMS avec respectivement 94.1% et 87.5% de sensibilité et 98.8% et 98.6% de spécificité;
- Les TDR BIOSYNEX^®^ COVID-19 Ag BSS et BIOSYNEX^®^COVID-19 Ag+ BSS peuvent être d´un grand intérêt dans les dépistages de masse et le diagnostic de SARS-CoV-2 dans des zones où la PCR demeure d´accès limité.

## Remerciements

Nous remercions les participants à la présente étude ainsi que nos équipes cliniques, de terrain et de laboratoire.

## References

[1] Organisation Mondiale de la Santé Situation épidémiologique du COVID-19 dans le monde et en Afrique au 21 juillet 202 Genève.

[2] Organisation Mondiale de la Santé Détection des antigènes à l´aide de tests immunologiques rapides pour le diagnostic de l´infection à SARS-CoV- 2020. Genève.

[3] Food and Drug Administration (2020). Potential for False Positive Results with Antigen Tests for Rapid Detection of SARS-CoV-2. Letter to Clinical Laboratory Staff and Health Care Providers.

[4] Centers for Disease Control and Prevention (2021). Overview of Testing for SARS-CoV-2 (COVID-19).

[5] Pilarowski G, Marquez C, Rubio L, Peng J, Martinez J, Black B (2020). Field performance and public health response using the BinaxNOW TM Rapid SARS-CoV2 antigen detection assay during community-based testing: clinical infectious diseases. Clin Infect Dis.

[6] Mbopi-Keou FX, Pondi JE, Sosso MA (2020). COVID-19 in Cameroon: a crucial equation to resolve. Lancet Infect Dis.

[7] Health Information and Quality Authority (2021). Public health measures and strategies to limit the spread of COVID-19: antigen testing in asymptomatic individuals in community settings.

[8] Okoye NC, Barker AP, Curtis K, Orlandi RC, Snavely EA, Wright C (2021). Performance Characteristics of BinaxNOW COVID-19 Antigen Card for Screening Asymptomatic Individuals in a University Setting. J Clin Microbiol.

[9] Charles Embola (2021). AFCON 2022: CAF to entrust COVID-19 Testing to an Independent Body.

[10] BIOSYNEX (2021). BIOSYNEX COVID-19 Ag+ BSS: SARS-CoV-2 antigen detection test.

[11] Lim C, Wannapinij P, White L, Day NPJ, Cooper BS, Peacock SJ (2013). Using a Web-Based application to define the accuracy of diagnostic tests when the gold standard is imperfect. PLoS One.

[12] Landis JR, Koch GG (1977). The measurement of observer agreement for categorical data. Biometrics.

[13] Jung C, Levy C, Varon E, Biscardi S, Batard C, Wollner A (2021). Diagnostic accuracy of SARS-CoV-2 (Antigen Detection Test in Children: a real-Life Study). Front Pediatr.

